# Convergent capture of retroviral superantigens by mammalian herpesviruses

**DOI:** 10.1038/ncomms9299

**Published:** 2015-09-24

**Authors:** Amr Aswad, Aris Katzourakis

**Affiliations:** 1 Department of Zoology, University of Oxford, Oxford OX1 3PS, UK

## Abstract

Horizontal gene transfer from retroviruses to mammals is well documented and extensive, but is rare between unrelated viruses with distinct genome types. Three herpesviruses encode a gene with similarity to a retroviral superantigen gene (*sag*) of the unrelated mouse mammary tumour virus (MMTV). We uncover ancient retroviral *sags* in over 20 mammals to reconstruct their shared history with herpesviral *sags*, revealing that the acquisition is a convergent evolutionary event. A retrovirus circulating in South American primates over 10 million years ago was the source of *sag* in two monkey herpesviruses, and a different retrovirus was the source of *sag* in a Peruvian rodent herpesvirus. We further show through a timescaled phylogenetic analysis that a cross-species transmission of monkey herpesviruses occurred after the acquisition of *sag*. These results reveal that a diverse range of ancient *sag-*containing retroviruses independently donated *sag* twice from two separate lineages that are distinct from MMTV.

## Abstract (web summary)

Horizontal gene transfer from retroviruses to mammals is rare between unrelated viruses. Here the authors show the convergent acquisition by herpesviruses of a virulence gene of ancient retroviruses, which occurred at least twice from different donor lineages, to distinct herpesviruses that infect mammals.

## Graphical abstract

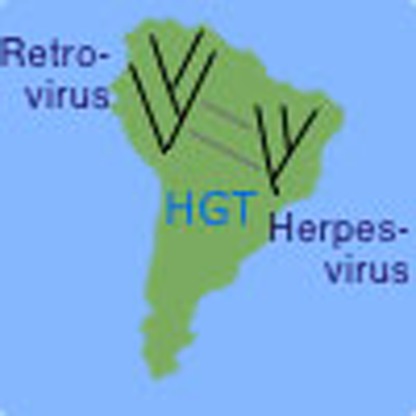

Superantigens (*sags*) are unique virulence genes capable of eliciting a major immune response by nonspecific T-cell stimulation^1^,^2^. Ordinarily, antigenic processing and presentation by major histocompatibility complexes (MHCs) is required before an immunogenic molecule can provoke T-cell recognition. *Sags* are unusual in their ability to crosslink antigen-presenting cells and T-cells by directly binding to MHC class II and T-cell receptors (TCRs) simultaneously^1^. Their affinity to a diverse range of TCRs results in large numbers of activated T-cells, causing a cytokine storm that can lead to potentially fatal toxic shock syndrome. *Sags* have also been implicated in a spectrum of unrelated syndromes, including sepsis, Kawasaki's disease, Scarlet Fever and autoimmune disorders^3^. Unrelated to bacterial *sag*, viral *sags* are also known, most notably in the mouse mammary tumour virus (MMTV)^4^, a retrovirus with an RNA genome. The integrated proviral genome encodes the main retroviral genes *gag*, *pro*, *pol* and *env*, as well as flanking long terminal repeats (LTRs) that are created during reverse transcription. LTRs are an integral part of retrovirus replication but are ordinarily non-coding. In MMTV, the majority of the *sag* gene is found within the U3 region of the LTR^4^. Multiple *sag* mRNA promoters have been described that give rise to different transcripts beginning in the U3/R junction in the 5′ LTR^5^,^6^,^7^, the *env* gene^8^ and an internal 3′ LTR U3 in *sag*^9^.

The *Herpesvirales* are a large, well-characterized and extremely diverse order known to infect amphibians, reptiles, fish, birds and mammals^10^. Humans are susceptible to eight different herpesviruses that are responsible for a number of diseases. These include, but are not limited to, oral and genital lesions, varicella (chicken pox), Burkitt's lymphoma and Kaposi's sarcoma^11^. Herpesviruses have large (over 100 kb) dsDNA genomes normally flanked by repetitive regions and blocks of conserved coding regions that vary between subfamilies. Gene content among herpesviruses is highly comparable, with the majority of lineage-specific genes existing towards the termini^10^. Members of the genus *Rhadinovirus* have a notably high number of host-derived genes, particularly in the region between the gene for dUTPase and the minor capsid such as the cellular genes for CD59 that in hosts is involved in complement regulation, and the proinflammatory cytokine interleukin (IL)-17. Also in this region, *sag*-like genes have been identified in *Saimirine Herpesvirus 2* (SaHV2) and its sister taxon *Ateline Herpesvirus 3* (AtHV3) that infect squirrel and spider monkeys, respectively^12^,^13^. A similar gene is also found in the genome of *Rodent Herpesvirus peru* (RHVP)^14^, a rhadinovirus of the pygmy rice rat (*Oligoryzomys microtis*).

We do not know the source of *sag-*like genes in these herpesviruses, nor do we know their evolutionary relationship to MMTV *sag*. However, because MMTV and herpesvirus *sags* are known to exhibit sequence similarity, we hypothesized that they are homologous as a result of horizontal gene transfer (HGT) from *sag*-containing retroviruses. Although HGT is prevalent among viruses in general, it is predominantly a phenomenon limited to those with comparable genomes. Virus-to-virus HGT between unrelated groups, such as between RNA and DNA viruses, is rare and not fully understood; however, there have been an increasing number of documented cases in recent years^15^,^16^.

Since MMTV is the only extant *sag*-containing retrovirus identified to date, we set out to investigate whether ancient retroviruses related to MMTV may have possessed *sag*. Viruses of every genome type and replication strategy are known to occasionally integrate into the germline genome of their host and can thereafter be inherited along with the host gene repertoire^17^,^18^,^19^. These endogenous retroviruses (ERVs) can eventually reach population fixation, and drift at the host neutral evolutionary rate. ERVs remain recognizable for millions of years after endogenization and can be used as a ‘fossil record' of past infections to understand viral evolution^17^,^18^. Our strategy was to use ERVs to determine the number of times *sag* was acquired by herpesviruses, and from how many retroviral donor lineages. We also aimed to characterize the relationship between endogenous retroviral *sags*, MMTV *sag* and herpesvirus *sags*, as well as the pattern of selective pressures involved in their respective evolutionary histories. To tackle these goals, we collected ancient retroviral data by mining ERVs from mammalian genomes. We then use these ERVs to reconstruct the history of this unique virulence gene, by synthesizing evidence from phylogenetics and bioinformatic techniques under an evolutionary framework.

To date, *sag-*containing ERVs have only been reported in a small number of genomes (for example, rat and deer mouse^20^); however, our systematic search reveals that there are numerous *sag-*like sequences in at least 20 different mammals. Through a series of phylogenetic analyses, we demonstrate that herpesvirus *sags* originate from a large, previously undescribed clade of betaretroviruses. Through an analysis of selection we demonstrate that these ancient *sag*-like genes were functional. The trees show that at least two HGT events occurred from different retroviral lineages that are both distinct from MMTV, and we provide estimates for the timing of these events. Our results show that, although gene transfer between unrelated viruses is rare, it can occur as a part of the evolutionary arms race in a convergent evolutionary manner.

## Results

### Characterization of the herpesvirus *sag* loci

**Figure 1:**
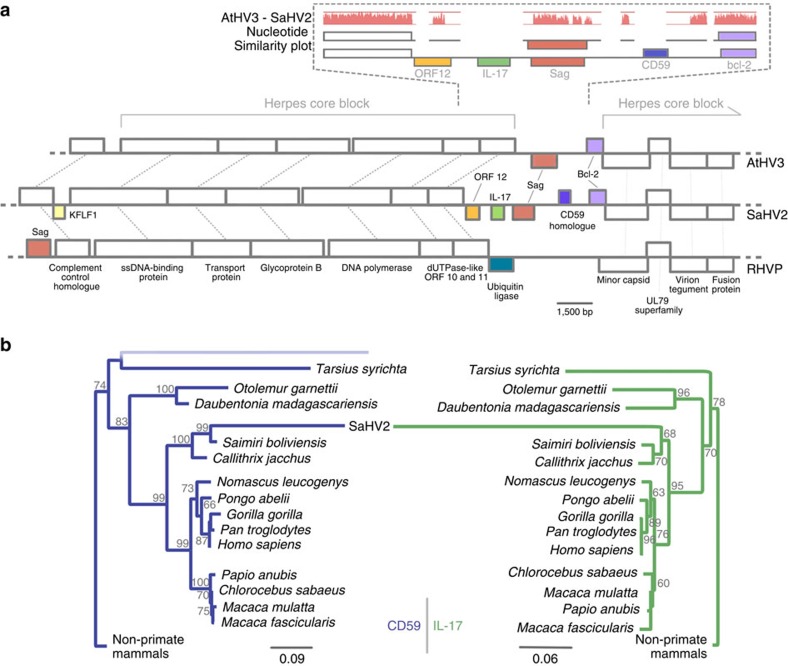
The herpesviral *sag* locus. (**a**) Cartoon of a partial genome alignment of SaHV2, AtHV3 and RHVP, showing conserved gene blocks in blank boxes and lineage-specific genes in colour, labeled according to product name. The figure also shows an alignment for SaHV2 and AtHV3 of the region between ORF11 and the minor capsid gene. The red graph depicts the percentage nucleotide-level similarity shared by the viruses. (**b**) Maximum-likelihood phylogenies of host IL-17 and CD59 from primates including a viral homologue in SaHV2. SaHV2 independently acquired these genes after diverging from AtHV3. The gene for CD59 in particular, appears to have been captured from the squirrel monkeys, which are the current host of the virus. Only the primate tips have been made visible. The scale bar represents the number of nucleotide substitutions per site. The results of 1,000 nonparametric bootstrap replicates are indicated at each node.

To determine whether herpesvirus and MMTV *sags* share an evolutionary history, we needed to first establish the evolutionary history of the gene among the herpesviruses. We conducted an analysis of the herpesvirus genomes to determine whether the three herpesvirus *sag* loci are syntenic, which would strongly suggest that their similarity is the result of common ancestry. It is straightforward to rule this out for RHVP *sag,* which is situated at a different locus to the other two herpesviruses (Fig. 1). The region between the dUTPase and minor capsid genes in SaHV2 and AtHV3 both share *sag* and *bcl-2* in a collinear manner, despite the presence of the genes for ORF12, Il-17 and CD59 in SaHV2 (Fig. 1). While ORF12 is absent in AtHV3, sequence similarity to SaHV2 at that locus is maintained, indicating that it was a shared gene in their ancestor that later degraded in AtHV3 (Fig. 1). This is not the case for the genes for IL-17 and CD59, where no sequence similarity is evident, demonstrating that they were acquired after the divergence of SaHV2 and AtHV3. Indeed, phylogenetic evidence shows that the genes in SaHV2 are closely related to the squirrel monkey homologues, which is the current host of SaHV2. Thus, these results show that besides *sag* the region between the genes for dUTPase and minor capsid in the SaHV2-AtHV3 ancestor included ORF12 and *bcl-2*. Whereas *bcl-2* persisted in both viruses after speciation, ORF12 was only maintained in SaHV2, which thereafter also acquired genes encoding IL-17 and CD59 (Fig. 1).

### Survey and alignment construction of *sag*-containing ERVs

**Figure 2:**
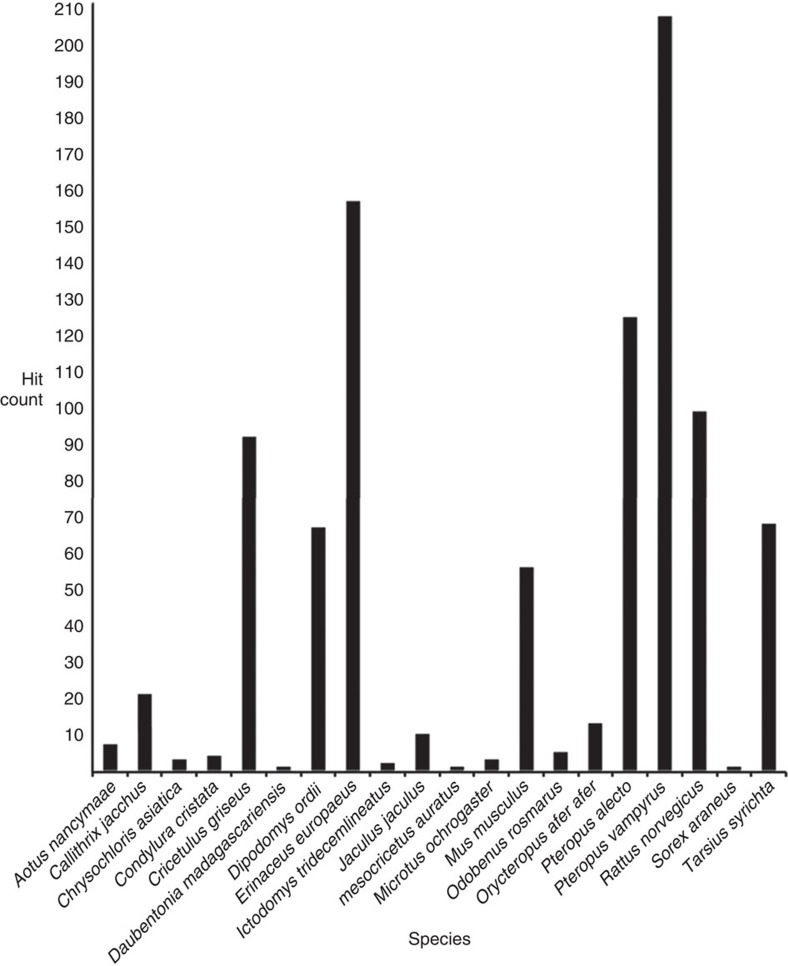
Summary of BLAST *sag* hits in mammalian genomes. The histogram shows the number of tBLASTn hits per mammalian genome to any of the four viral *sag*s. A conserved 100 amino-acid region of the *sag* sequences of SaHV2, AtHV3, RHVP and MMTV was used as a query. The mouse, rat, Chinese hamster and hedgehog data represent multiple individual genomes. The data were obtained from the NCBI whole genome shotgun (WGS) database.

We surveyed mammalian genomes to search for evidence that retroviruses with *sag* have existed in the past. We used BLAST with the herpesviral *sag* open reading frames and a representative MMTV *sag* transcript sequence as queries to search for similarity in NCBI genome databases (Fig. 1 and Supplementary Fig. 1). To discard false positives and sequences that are too divergent to be aligned, we applied stringent shortlisting criteria to the results (see Methods). The search revealed many ERVs with *sag* similarity, representing sequences from the genomes of a diverse set of 20 species of mammal (Fig. 2). All of these *sags* were betaretroviral in origin and were closely related to MMTV. We also searched for betaretrovirus ERVs using MMTV polymerase (Pol) as the query, to identify those closely related to MMTV that did not show *sag* similarity. To investigate the evolution of herpesvirus *sag* acquisition, we used the collected sequences to conduct a series of phylogenetic analyses that included data from both sets of searches.

**Figure 3:**
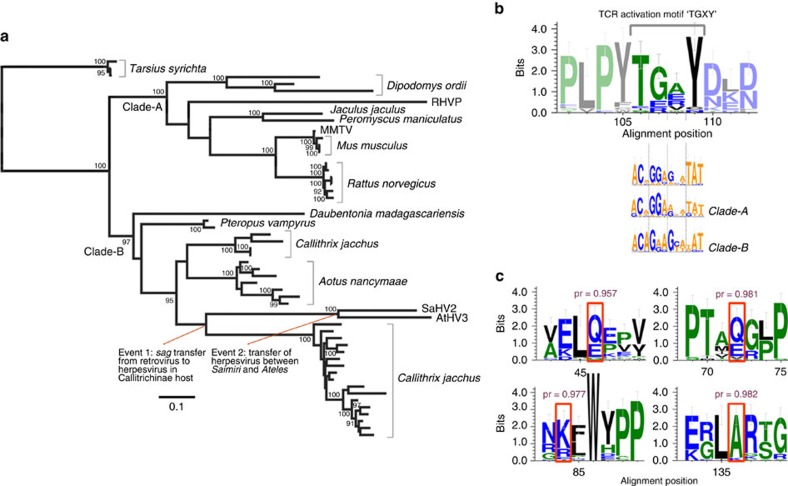
Evolutionary history and selection analysis of *sags.* (**a**) A phylogeny of *gag, pro, pol, env* and *sag* genes. Clade-A shows the rodent ERVs, and RHVP, while clade-B is the predominantly primate clade that also contains AtHV3 and AtHV2. The rooting was determined by reconstructing a tree from an alignment of *pol* from sequences included in this tree as well as 11 outgroup sequences. The scale bar shown represents the number of amino-acid substitutions per site and posterior probability values above 90% are indicated at each node. (**b**) WebLogo of a region of the alignment used to reconstruct the tree in **a**, including the underlying codons on the first line. The region shown is the crucial TCR activation motif. The subsequent lines show the codons for the same region using only clade-A or clade-B sequences, including a larger taxon set compared with the representative subset used for the tree. (**c**) Results of the Branch-Site test of positive selection, assessing the likelihood of positive selection at individual sites of the branch leading to SaHV2/AtHV3. The WebLogo was generated using the expanded clade-A alignment. The highlighted sites have a d*N*/d*S*>1 with a significant posterior probability, denoted as pr.

We employed a phylogenetic strategy that would allow us to both investigate *sag-*less sequences and also mitigate the constraints of low sequence similarity among various *sags* (only a short region (∼80 amino acid (aa)) could be aligned across all species (Supplementary Data 1)). Conserved regions of all four retroviral genes plus *sag* were concatenated in a ‘master' alignment of 138 sequences including the three herpesvirus *sag*s, MMTV, related ERVs with or without *sag*, and a number of other retroviruses such as JSRV, ALV and HERV-K that are known outgroups to MMTV (Supplementary Fig. 2). Because some sequences did not include all genes (Supplementary Table 1), the proportion of gaps in the master alignment was prohibitive for meaningful phylogenetic reconstruction. For this reason, we constructed a concatenated alignment of all genes excluding many sequences that were predominantly gapped (Supplementary Fig. 2) except for those of the monkey *sags* that are the most closely related to SaHV2 and AtHV3 *sag* (Fig. 3). The subset of these sequences that also contained *pol* was additionally aligned to the outgroup sequences to identify the correct root of the main concatenated tree (Supplementary Fig. 3). We also reconstructed a tree from the *sag*-only alignment to evaluate the placement of sequences omitted from the main tree, and a *pol*-only tree to assess the relationship of *sag-*containing ERVs to closely related non-*sag* ERV.

### Reconstruction of the evolutionary history of *sag*

The concatenated alignment revealed that there are two major lineages of *sag*—one belonging to retroviruses that primarily infect rodents (designated ‘clade-A'), and a second mainly primate-infecting ‘clade-B' (Fig. 3). Importantly, the *sags* of primate-infecting herpesviruses (SaHV2 and AtHV3) are most closely related to primate retroviral *sags*. We identified three independent lineages in the genome of *Callithrix jacchus* and *Aotus nancymaae,* which are not orthologous, despite the hosts having only recently speciated (∼20 million years ago (mya)). Together, they form a robust monophyletic group that is consistent with a single-capture event of *sag* from a new world monkey retrovirus to the ancestor of the two new world monkey herpesviruses. The RHVP *sag* does not cluster with the monkey herpesvirus *sags,* indicating a separate gene-capture event. Instead, RHVP *sag* is more closely related to rodent retroviruses, resolving as a sister taxon to *J. jaculus* and *P. maniculatus*. Both herpesvirus *sag* lineages resolve within a clade of retroviruses to the exclusion of other retroviral sequences with high posterior probability, indicating that the direction of transfer was from retrovirus to herpesvirus. Furthermore, the tree shows with 100% probability that both of the herpesvirus *sag* lineages are independent of MMTV *sag*, which is the only other extant *sag*-containing virus.

**Figure 4:**
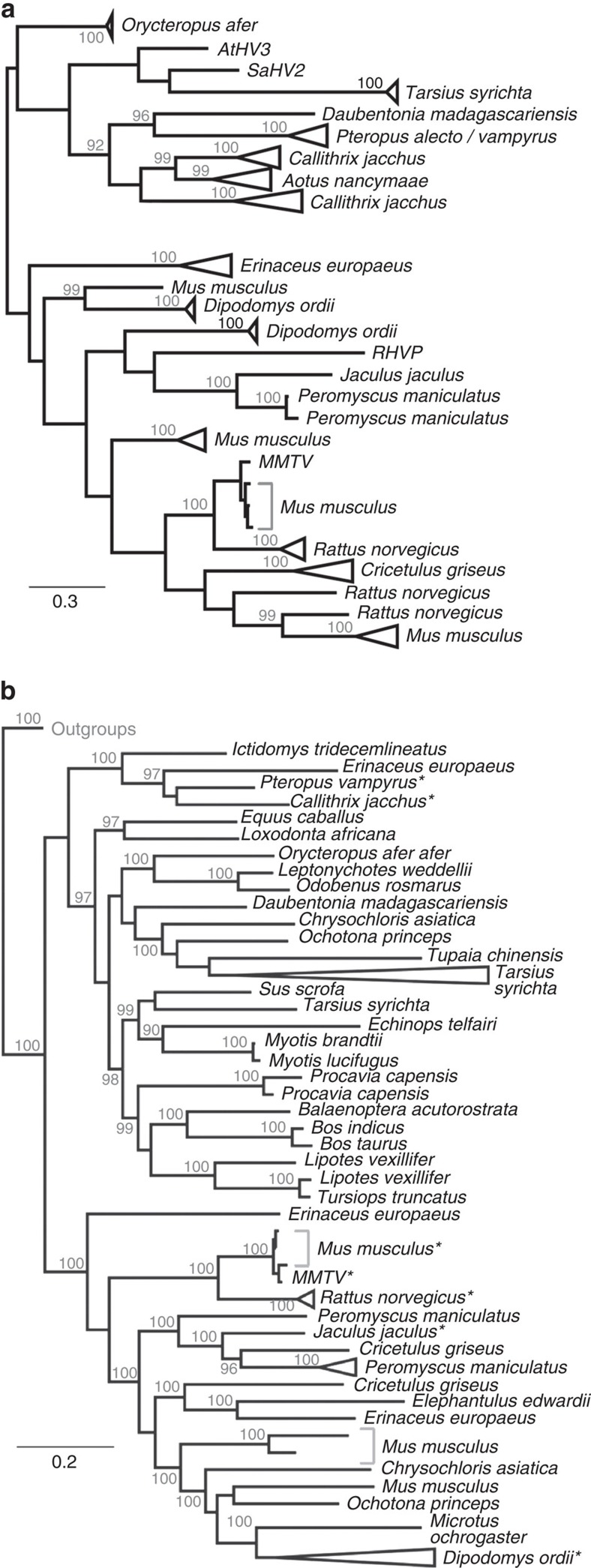
Phylogenetic tree of MMTV-like polymerase and *sag* sequences. (**a**) The Bayesian phylogenetic tree shown was reconstructed using only the *sag* partition of the alignment. Albeit with lower support for deep nodes, the *sag*-only tree recovers the distinction between predominantly rodent- and primate-infecting viruses. The tree reveals that there are three independent lineages of rat ERVs, four lineages of mouse and two separate kangaroo rat clades. The scale bar shown represents amino-acid substitutions per site, and posterior probability values above 90% are indicated at each node. (**b**) The Bayesian phylogenetic tree shown was reconstructed using only the reverse transcriptase and integrase (RT and INT) partition of the alignment. Both clades A and B are observed as with the Fig. 3 and *sag*-only tree in the upper panel. In addition, the RT–INT tree shows that clade-A is closely related to a large and diverse group of *sag-*less mammalian ERVs. Starred tips indicate a *pol* of an ERV also included in the phylogenetic analysis of a concatenated alignment of *gag, pol, env* and *sag*. The scale bar shown represents amino-acid substitutions per site, and posterior probability values above 90% are indicated at each node.

Not all of the *sag* genes identified were part of full-length ERVs (Supplementary Table 1). Most were found as ‘solo-LTRs', which are the result of recombination of LTRs that leads to the deletion of the rest of the ERV. Overall 490/527 of the shortlisted *sag*-containing contigs did not return BLAST hits to other retroviral genes (using MMTV *gag, pol* and *env* as queries), indicating that they were solo-LTRs. Therefore, the separate *sag* alignment allowed the inclusion of more retroviral sequences, some of which were excluded from the concatenated alignment because of the absence of any other genes. Because it is based on a much smaller alignment of a highly divergent gene, the topology of this tree is less reliable and exhibits poor support overall. Nevertheless, the *sag*-only tree does recover the distinction between the predominantly rodent- and primate-infecting viruses, albeit with lower support for deep nodes (Fig. 4). The *sag*-only tree shows evidence for multiple invasions by MMTV-like retroviruses of the same host species; there are three independent lineages of ERVs in *Rattus norvegicus*, four lineages in *Mus musculus* and two separate *Dipodomys ordii* clades (Fig. 4). The *sag*-only tree also shows a larger subset of the ∼300 *Pteropus sp. sag*-like BLAST hits (Fig. 2) that indicate a history of intragenomic proliferation. As in the case of the multilineage *sag*s of mouse, rat and kangaroo rat, there are two distinct lineages in *C. jacchus* as well as a lineage in *A. nancymaae* (Fig. 4).

The *pol* tree (Fig. 4) allows us to reliably identify the phylogenetic placement of *sag*-containing ERVs identified in this study with respect to other retroviruses. It was possible to include ERVs that were identified as being closely related to MMTV, but did not contain recognizable *sag* according to our similarity cutoff. This tree also shows that none of the *pol* sequences from *sag-*containing ERVs are more closely related to a non-betaretrovirus (for example, in the case that a recombination event occurred). The inclusion of known outgroup sequences in this tree confirms the distinction between clades A and B from which the separate herpesvirus *sag* acquisitions originate. Interestingly, the *pol* tree reveals that there is a diverse group of mammalian ERVs closely related to the *sag-*containing viruses. Most of these viruses did not contain detectable *sag* sequences according to our similarity threshold for consideration (Fig. 4), except for a lineage of *Tarsius syrichta* ERVs, which resolve at the root of the main phylogeny.

### Reconstructing the *sag* evolutionary timeline

**Figure 5:**
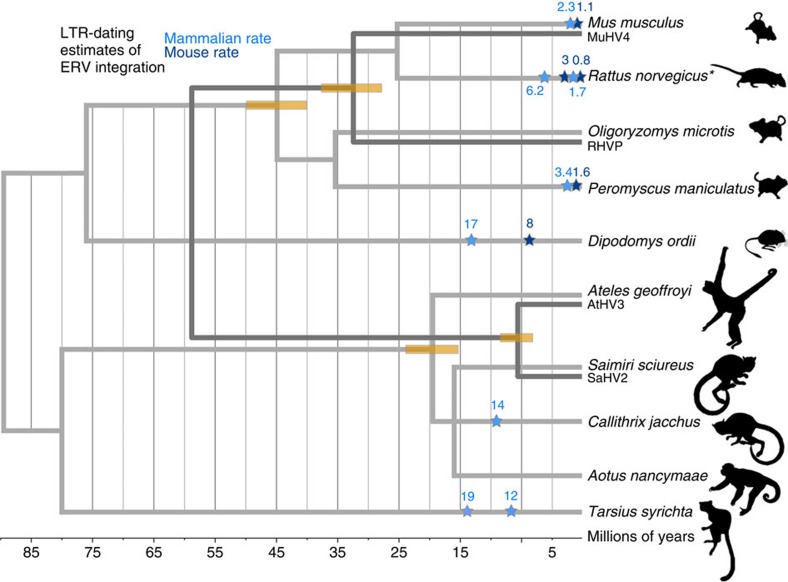
Evolutionary scenario of herpesvirus and retrovirus *sag.* The figure depicts time-calibrated phylogenies of Herpesviruses (dark grey) and mammalian hosts (light grey). Both trees are shown on the same axis and have been pruned down to the taxa relevant to this study. Each of the hosts for which *sag-*containing ERVs could be dated through LTR divergence is marked by stars that indicate the age estimate obtained using different neutral rates of evolution. Only the highest and lowest values for *R. norvegicus* are shown for clarity (denoted by an asterisk). The herpesvirus branches are drawn alongside the relevant host branch, to evaluate whether their respective divergence times are consistent with the null hypothesis of co-speciation. Yellow bars indicate the upper and lower bound age estimates. We cannot confidently reject a scenario of co-speciation for RHVP and MuHV4 since there is a known increase in the rate of evolution along this herpesvirus lineage, and the divergence date estimates are close to those of their hosts. The split between SaHV2 and AtHV3, however, is far younger than that of their hosts with no discernable rate increase. This discrepancy is consistent with cross-species transmission of the herpesviruses rather than co-speciation. The divergence date of the two monkey viruses therefore is a minimum bound for the acquisition of *sag*. This date (∼10 mya) is also similar to the integration date estimate of a closely related *sag* in the *C. jacchus* genome (∼14 mya), which supports the existence of exogenous retroviruses circulating in new world monkeys, with a maximum bound at the root of the host clade ∼25 mya.

Since retroviral LTRs are identical at the time of integration, their divergence from one another is used as a measure of their age when combined with estimates of the host neutral rate. We calculated the age of the ERVs on the basis of the average mammalian rate^21^ of 2.2 × 10^−9^ per site per year, revealing that sequences in clade B are relatively young. The rat, mouse and deer mouse ERVs are between 1.7 and 6.2 mya, whereas the marmoset and tarsier ERVs are estimated to be between 14 and 19 mya, respectively. Using the mouse evolutionary rate (4.5 × 10^−9^ per site per year)^22^, age estimates for clade-B are calculated to be between 0.8 and 3 mya. However, both sets of estimates should be interpreted as conservative minimum bounds for the integration date of the exogenous virus (Fig. 5) because they are averages over time and across the genome. Moreover, apart from mouse, a species-specific evolutionary rate has not been obtained for the other hosts.

We wanted to test whether the divergence of the herpesvirus is similar to their respective host divergence dates (that is, the age of the common ancestors of SaHV2-AtHV3 and RHVP-MuHV4 should be consistent with the age of the *Saimiri*-*Ateles* and *Oligoryzomys-Mus* divergences, respectively). We therefore reconstructed a time-calibrated phylogeny of rhadinoviruses using a relaxed log-normal molecular clock implemented in BEAST. We calibrated at previously identified co-diverging nodes (using host divergence dates^23^) that are outside the clades of interest, therefore enabling us to date the *sag*-containing herpesvirus splits without assuming co-divergence *a priori.* This revealed that the SaHV2-AtHV3 split occurred ∼10.6 mya (95% HPD: 8.4–13), while that of RHVP and its closest relative MuHV4 diverged∼37.5 mya (95% highest posterior density (HPD): 32.7–42.6; Fig. 5).

### Selection analysis of retroviral and herpesviral *sags*

The results from the phylogenetic analysis support the hypothesis that *sag* was co-opted by herpesviruses from retroviruses. If this is true, the retroviral donors must have possessed functional *sag,* and so we sought evidence for a history of purifying selection in the *sag-*like sequences that we identified. We conducted a maximum-likelihood-based analysis of the ratio of non-synonymous to synonymous (d*N/*d*S* or *ω*) using PAML^24^. We estimated average d*N/*d*S* along branches in the tree (the so-called branch models) by allowing every lineage to have its own *ω* (free-ratio test) or fixing the tree to a single estimate (one-ratio test). We also tested a number of two- and multi-ratio tests that did not return a significant improvement of likelihood over the one-ratio test (see Methods, Supplementary Table 2). Despite the fact that the majority of the sequences are neutrally evolving non-functional ERVs, the one-ratio test revealed *ω*=0.289, which is indicative of purifying selection. This means that their endogenization was relatively recent (which is consistent with the results of the LTR dating) and these sequences were likely functional in the past. This also indicates that the donor was most likely an infectious retrovirus rather than an integrated virus, which would have a d*N/*d*S* value much closer to one because of neutral drift in the host genome. Furthermore, we find that a known conserved motif—crucial for TCR activation^1^—is well conserved in the *sag*-like sequences of the ERVs investigated (Fig. 3). In sequences without the exact TGXY motif, conservation is, nonetheless, maintained at the nucleotide level, suggesting that the site has drifted since endogenization (Fig. 3).

To investigate whether adaptive changes occurred in *sag* during capture by herpesviruses, we implemented the branch-site model to test for positive selection at individual sites along the internal monkey herpesvirus branch. This revealed that four amino acids exhibited evidence for positive selection (*P*<0.0001, Fig. 3). For sites 46 and 72, we see either glutamine or glutamic acid in the majority of *sags*, both of which are polar but have changed in monkey herpesviruses to the small, non-polar glycine and valine, respectively (Fig. 3). Interestingly, amino acid 84 is a lysine in most sequences but has undergone a triple-site change in SaHV2/AtHV3 (AAA-CGC, relative to *C. jacchus*) to encode the similarly positively charged arginine, despite only needing a single-site change. This could indicate that this biochemical property is important here, despite being a positively selected site. A triple-site change at residue 136 for AtHV3/SaHV2 encodes lycine rather than alanine as in most *sags.* Positions 84 and 136 are also different in RHVP relative to the phylogenetically closest sequences, but neither change is equivalent to the change in SaHV2 and AtHV3, nor are they biochemically comparable. This could mean that these sites are under diversifying selection, or else could reflect adaptation to host-specific targets. Owing to the limitations of selection analyses, we consider these four sites as the minimum number of possible adaptive sites. This is because selection analyses cannot detect selective sweeps (where an adaptive site is adopted across the entire population).

While the principal drivers of these patterns of selection would have taken effect on *sag* in the exogenous virus, they could have continued post endogenization, if *sag* was co-opted by the host. For instance, endogenous *sag* expression in mice can be viewed as beneficial to the host since it reduces the spread of exogenous MMTV^25^. To look for post endogenization purifying selection, we examined orthologous loci to control for the signal of purifying selection that results from exogenous viral evolution but did not find any significant evidence of d*N/*d*S*<1. We identified orthologous *sag* sequences in the two bat species as well as in the different strains of mouse by aligning the LTRs and checking to see whether the host flanking sequence was identical. There were zero differences between the mouse sequences and none of the 25 orthologous *Pteropus* LTRs exhibited a d*N/*d*S* significantly different from 1 according to the likelihood ratio test with a *P* value threshold of 0.05 (Supplementary Table 3). Nonetheless, it is noteworthy that a search of the NCBI expression databases EST and TSA reveals hits to *sag* in the mouse (120), deer mouse (12), rat (3) and Chinese hamster (3) with an E value threshold of 0.00001, a minimum alignment length of 60 amino acids with no more than 20 mismatches.

## Discussion

We set out to reconstruct the unusual evolutionary history of viral *sags*, which exhibit sequence similarity despite belonging to unrelated viruses. In this study, we uncovered an unexpected diversity and richness of *sags* in the retroviral fossil record, offering us the necessary evolutionary context in which to reconstruct the shared history of herpesvirus and MMTV *sag.* The results conclusively demonstrate that the herpesviral *sag*s were the result of convergent HGT from ancient retroviruses related to MMTV. Our analyses reveal that this HGT occurred on at least two different occasions into the same herpesvirus genus, from two independent retroviral donors that are both distinct from MMTV.

The multiple origins of herpesvirus *sags* are consistent with both the polyphyly of *sag-*containing herpesviruses (Supplementary Fig. 4) as well as the fact that the RHVP *sag* locus is not orthologous to the other herpesvirus *sags*. This allows us to conclusively rule out a single-capture scenario in the ancestor of the three rhadinoviruses, all of which infect South American hosts. Indeed, a scenario where *sag* was transferred from retroviruses once does not explain why *sag-*containing herpesviruses are not monophyletic, and would also require multiple losses of *sag* in other herpesviruses, and at least one recombination event to explain why it is in a different location in RHVP.

We are able to reconstruct a timeline of events in the evolutionary history of *sag* by combining information from the *sag* phylogeny with host and herpesvirus speciation dates, as well as the dates of ERV integration (Fig. 5). It should be noted that this should only be considered an estimate of the time frame because of the inaccuracies of molecular clock dating. For example, the evolutionary rate can vary between different locations in the genome^26^, and is known to correlate with a number of other variables such as body size, lifespan, generation time and fecundity^27^, as well as varying between different lineages for specific genes^28^. Such variation could be particularly influential for the LTR dating because we could be applying estimates on the basis of averages to specific loci in different species (for example, using the mouse average rate for specific mouse loci or for loci in other rodents). However, this variation in the evolutionary rate is less problematic for both the host tree dates obtained from ref. 23 and our timescaled herpesvirus tree, which is calibrated using these dates. This is because of the observation that evolutionary rates are relatively homogenous across mammals when the estimates are obtained by averaging over many genes simultaneously^28^. With these caveats in mind, the lower bound estimate for the mouse-pygmy rice rat split is 45.1 my, which is close to the upper bound of the 95% confidence interval for the MuHV4-RHVP split (42.6 mya) obtained from our dating analysis. However, although the dates do not overlap exactly, this can be explained by the increased rate of evolution in the MuHV4-RHVP lineage that would result in an underestimated date. This observation has been previously made^29^ and is confirmed in our analysis by the long branches of MuHV4 and RHVP. Another reason for the discrepancy in ages is that the viral speciation could have occurred slightly after that of the hosts, since it would have continued to cross-infect for some time. We therefore cannot reject the hypothesis that RHVP and MuHV4 co-speciated with their hosts. We can therefore use the speciation of mouse and pygmy rice rat as an upper bound for the acquisition of *sag*, since the gene is absent in MuHV4.

On the other hand, the AtHV3-SaHV2 common ancestor is estimated to be much younger than the split of their hosts at 10.6 mya compared with 24.5 mya. Moreover, we did not detect an elevated evolutionary rate that could explain the difference nor have any other previously published studies^29^,^30^. This means that one of the two viruses arose from a cross-species transmission several million years after the hosts diverged. Because the *sag* genes in both viruses are at the same locus (Fig. 1) and are phylogenetically most similar to each other, they most likely originated from a single acquisition in their common ancestor. However, since the genes are located in a gene-capture hotspot, we cannot rule out the possibility that they appear syntenic by coincidence, and that their phylogenetic similarity is because of convergent evolution. To examine the latter possibility, we re-estimated the phylogeny after removing the amino-acid positions that we identified as positively selected in herpesviruses (Fig. 3), in case these sites led them to appear artificially more similar (Supplementary Fig. 5). Nonetheless, the viruses still resolve as a monophyletic group, which is consistent with the single-capture hypothesis.

It will be interesting to see whether future studies identify retroviral lineages that split the monophyly of the herpesvirus *sags*, which would mean that the convergent capture of *sag* occurs more often than our findings demonstrate. Whether there were one or two HGT events, multiple independent integrations of non-orthologous ERVs in both *Aotus and Calithrix* species indicate that they had a single infectious retroviral ancestor, which is also ancestral to the SaHV2/AtHV3 clade (Fig. 3). This means that the retroviral donor to the monkey herpesviruses was also infectious, which is consistent with the finding of low d*N/*d*S* values along the monkey herpesvirus *sag* internal branch. Moreover, the fact that *sag* is absent from any Old World primate ERVs suggests that the retrovirus crossed into New world monkeys after their arrival in South America in the late Eocene/Early Oligocene^31^. Together, these data support the existence of an infectious retrovirus circulating among and transmitting between monkeys in South America, leaving endogenous counterparts in multiple host and herpesvirus genomes.

The search for past evidence of *sag* in the retroviral fossil record revealed at least 20 separate lineages of ERV from eight mammalian orders with *sag* similarity in their LTR (Fig. 4). While our shortlisting criteria are a conservative estimate of the past diversity of *sag-*containing retroviruses, the number of taxa included in the trees and the long branches between clades is strong evidence of the diverse and widespread nature of *sags* in ancient betaretroviruses. The vast majority of ERVs will eventually succumb to deletion because of illegitimate recombination between the LTRs. For example, the number of solo-LTRs in the human genome far outnumbers the count of full-length ERVs^32^. Being in the LTR, *sag* is therefore more likely to be preserved in the fossil record, and this is reflected in the results of our search. The LTRs of retroviruses tend to be poorly conserved beyond closely related clades^33^, since there is little selective pressure to maintain a particular sequence. However, the fact that the LTRs encode *sag* in MMTV-like betaretroviruses means that they are conserved across a more diverse range of retroviruses. Therefore, besides revealing the history of herpesvirus *sag* acquisition, the *sag* tree also represents an analysis of LTR evolution in deep time that is based on a sequence alignment, rather than the alternative methods that are necessary to circumvent the difficulty of aligning LTRs^34^.

Herpesviruses typically infect hosts indefinitely, and move from latent to lytic replication during periods of immunosuppression. A host expressing *sag* mRNA is more likely to be in an immunocompromised state that is the ideal environment for *sag* gene transfer in an activated herpesvirus. For the gene transfer to take place, a herpesvirus would have likely infected a host with a high retroviral load, which could consequently lead to the accidental integration of the retrovirus followed by the formation of a solo LTR. Experimental evidence establishing the ability of retroviral integration into herpesviruses was demonstrated over a decade ago, where *Reticulendotheliosis virus* (REV) and Marek's Disease Virus (MDV) were co-infected both *in vitro* and *in vivo*^35^. More recently, a number of studies have shown that the naturally occurring REV LTR in field strains of MDV directly influence MDV expression and pathogenicity^36^. The phenomenon is not limited to herpesvirus as recipients, nor are retroviruses their only donor. The Fowlpox Virus is also known to have recombined with REV^37^, and the small single-stranded DNA adeno-associated virus was the source of gene captured by *Human Herpesvirus 6* (ref. 38). A common biological mechanism in these examples is that the recipients are large DNA viruses, while the donors are small viruses with an established DNA integration mechanism. The high chance of illegitimate recombination between LTRs, leading to the loss of all but a solo-LTR, explains the absence of the rest of the virus in herpesviruses and is the mechanism that has been demonstrated in the case of MDV^39^. Alternatively, although we identified a low d*N/*d*S* value along the internal herpesvirus branch, it is possible that the acquisition did not occur during co-infection by retroviral integration followed by solo-LTR formation. It could be that a host-integrated retrovirus expressed *sag* mRNA that was then reverse-transcribed and integrated by recombining with a retroelement LINE or SINE, although no evidence of any such element was detected using RepeatMasker, nor did we identify a poly-A tail that is characteristic of mRNA.

The convergent acquisition of the same gene by herpesviruses of the same genus suggests a shared evolutionary mechanism. MMTV-like retroviruses have donated *sag* to retroviruses, which implicitly reveals a history of retrovirus–herpesvirus co-infection in two different mammalian orders. The immunomodulatory benefit offered by *sag* explains its tendency to be captured as part of the perpetual arms race that viruses and hosts are engaged in; herpesviruses tend to capture sequences with immunomodulatory functions^40^. In the case of the monkey herpesviruses, the gene was maintained after speciation, which reflects the selective advantage it may have offered at a time when new world monkeys were rapidly diversifying in South America.

The finding that *sag* transfer between unrelated viruses was convergent could indicate that rhadinoviruses and retroviruses are particularly amenable to co-infection, which would explain the recurrence of an otherwise extremely rare event. In the case of *Human Herpesvirus 8*, the aetiological agent of Kaposi's sarcoma, symptoms are almost exclusive to HIV patients, which is also a retrovirus. The data have also revealed that *sag* genes are widespread in ancient mammalian betaretroviruses, suggesting that many mammals are potential reservoirs of hitherto unknown *sag-*containing viruses, potentially offering immediate applications in epidemiological surveillance. Furthermore, our findings could facilitate future surveillance efforts in anticipating similar gene acquisitions that might drastically change the recipient virus' pathology. Indeed, we do not fully understand what drives the differences in pathogenicity of many herpesviruses, both in the course of a lifetime infection and in different individuals.

Host and virus evolutionary rates differ dramatically, an imbalance favouring viruses that quickly adapt to host defenses. One compensation mechanism employed by hosts is the occasional evolutionary shortcut in the form of viral gene capture, bypassing the need for slow molecular adaptation by re-purposing viral genes that have already undergone the necessary evolution^25^. Interestingly, the endogenous counterpart of MMTV in the mouse offers an advantage against exogenous MMTV. Expression of *sag* during thymic development results in deletion of T cells that are *sag*-responsive, reducing the viruses' capacity to proliferate^41^. Viral gene capture by hosts may facilitate viral endogenization in general but is especially likely in the case of *sag*^25^. This is because the endogenous expression of *sag* is itself a rapid solution to the problems it causes, and the selective pressure imposed on *sag*-less individuals mitigates the negative effect of host *sag* expression. The herpesvirus capture of *sag* is exploiting the same evolutionary advantage as the host for the opposite reason—as a countermeasure to host immunity. Moreover, the fact that *sag* acquisition is convergent indicates a common evolutionary mechanism, which is distinct from that involved in host gene capture. Herpesviruses are exploiting the product of the evolutionary arms race in which retroviruses developed *sag*. In this way, the virus receives a gene with the specific function of counteracting host defenses, but without incurring the cost of incremental molecular evolution.

## Methods

### Sequence collection and phylogenetic reconstruction

We conducted similarity searches by tBLASTn, using MMTV amino-acid sequences of Sag and Pol as a query against the whole genome shotgun database (Genbank Accession NP_056884.1 and NP_056880.1, respectively). In all, 19/20 of the genomes identified in this study were obtained from the database as it was in November 2013, with the later addition of the ERVs in the *Aotus nancymaae* genome that was released during manuscript preparation. The search using MMTV Pol retrieved many distantly related ERVs. To delimit the sequences to those closely related to MMTV, a neighbour-joining tree was reconstructed using the reverse transcriptase domain of 4,750 sequences. We discarded all but a subset of ∼100 sequences from the MMTV-like clade and three known outgroups (Avian Leukosis Virus, Human ERV-K108 and JSRV). We also included eight ERV sequences that were closely related to these outgroups: *Rattus norvegicus, P. alecto* x2*, Ovis aries, A. nancymaae, Nomascus leucgenys and M. musculus*. We manually curated the shortlist by re-examining the whole genome shotgun database, using query sequences distributed across the tree. These searches were also carried out against individual species, as the BLAST scores vary according to the size of the target database, and erroneously excluded ERVs were reintroduced into the list. Each gene was aligned automatically with MUSCLE with manual adjustment and only conserved regions were used for further analysis. The *sag* sequences used were shortlisted according to another round of BLAST searching using the conserved region from each of MMTV, SaHV2, AtHV3 and RHVP as a query. We shortlisted *sag* sequences that aligned to at least 80% of any of the queries, with at least 45% identical positions and no more than 45 mismatches. The short list was then reduced to a representative subset using a guide tree, discarding highly similar sequences and favouring contigs that contained the whole ERV. Bayesian phylogenetic analysis was performed using parallel MrBayes 3.2.2. For each tree, two independent MCMC chains were run for 50 million generations with a 25% burn-in. The tree depicted in Fig. 3 was reconstructed from a concatenated amino-acid alignment of *gag, pro, pol, env* and *sag*. We could not include the outgroup set in this concatenated alignment because of the absence of any retroviral genes in solo-LTRs and herpesvirus *sags.* Therefore, to determine the rooting of the tree, we reconstructed a diagnostic phylogeny from an alignment of the outgroup with a subset of the concatenated alignment that excluded the solo-LTRs and herpesvirus sags. This tree revealed the *Tarsius syrichta* ERVs as the most basal sequences with a posterior probability of 100, allowing us to correctly root the Fig. 3 tree.

Prottest 3.4 was used to determine the best evolutionary model, which was JTT+G for Gag, Env and Sag; RTREV+G for Pro and WAG+G for Pol.

### LTR dating and BEAST analysis

The date of integration was estimated for paired LTRs from ERVs included in the tree reconstructed from the concatenated alignment. The LTRs were identified using BLASTn to search one half of the ERV against the other. The maximum likelihood (ML) distance of aligned LTRs was obtained using BASEML^24^, and dates were calculated by dividing half the genetic distance by the host neutral rate of evolution. We reconstructed a maximum clade credibility Bayesian phylogenetic tree in BEAST from a concatenated alignment of six core genes (terminase, large tegument, uracil-DNA glycosylase, kinase, capsid protein and helicase) using the GTR+Γ substitution model and a relaxed uncorrelated log-normal molecular clock model. As in previous studies^29^, we calibrated the tree using the divergence date of *Ovine Herpesvirus 2* from *Porcine Lymphotropic Herpesvirus 2*, their divergence from *Equine Herpesvirus 2*, and the split between Old World primate Lymphocryptoviruses from everything else. On the basis of the split of their hosts, the respective values in millions of years used were as follows: ruminant-pig divergence (71.6), artiodactyl-perissodactyl divergence (87.3) and primate–ungulate divergence (98.9)^23^. Only a pruned version of the BEAST tree is shown in Fig. 5; the full tree can be found in Supplementary Fig. 6.

### Selection analysis

We analysed d*N/*d*S* values along branches in the tree using CODEML^24^. We estimated *ω* across the whole tree assuming the same value for all branches (one-ratio test) or allowing each branch a separate value (free-ratio test). The free-ratio model explained the data significantly better than the one-ratio (*P=*0.006 Fig. 3) by a likelihood ratio test where the *χ*^2^ value was equal to twice the difference of the log likelihoods with 49 degrees of freedom. The free-ratio *ω* estimate for the monkey herpesvirus internal branch (0.461) was higher than the tips (0.238 and 0.307). These values represent an average along the branches and should be interpreted only as a guide in the exploration of selection patterns in the tree. We were interested in whether this reflected an episode of positive selection at the time of *sag* acquisition, and whether a convergent change could be detected in RHVP. To investigate this and address the problem of overparameterization, a number of models were implemented that group branches into a small number of categories. This included distinguishing herpesviruses from retroviruses, clade-A from clade-B, and tips from internal branches (Supplementary Table 2). We also implemented the branch-site test for the internal branch of SaHV2 and AtHV3. Significance was determined by a log likelihood ratio test where the null hypothesis is that d*N*/d*S*=1 (*P*<0.0001). Because selective sweeps cannot be detected, our findings are likely to be an underestimate of the positively evolving sites in *sag.* Nonetheless, the Bayes Empirical Bayes analysis indicated five sites evolving under positive selection. One of the sites detected is tentative because of low certainty of the alignment at that position, and the remaining sites were visualized using a WebLogo (Fig. 3). The likelihood scores of estimated pairwise d*N*/d*S* values of *Pteropus* orthologues were compared against the score of fixing the d*N*/d*S* to one using an LTR.

## Additional information

**How to cite this article:** Aswad, A. & Katzourakis, A. Convergent capture of retroviral superantigens by mammalian herpesviruses. *Nat. Commun.* 6:8299 doi: 10.1038/ncomms9299 (2015).

## Supplementary Material

Supplementary Figures, Supplementary Tables and Supplementary References Supplementary Figures 1-6, Supplementary Tables 1-3 and Supplementary References

Supplementary Data 1 Fasta file of the sag-only alignment used for phylogenetic reconstruction in figure 4. Note that the shorter sequences represent the conserved ~80aa region that can be aligned across all taxa.
