# Supplementary material for: Convergent capture of retroviral superantigens by mammalian herpesviruses
Source: Nat Commun. 2015 Sep 24;6:8299. doi: 10.1038/ncomms9299 (PMC4667437; doi:10.1038/ncomms9299)
Supplement: Supplementary Figures, Supplementary Tables and Supplementary References — Supplementary Figures 1-6, Supplementary Tables 1-3 and Supplementary References [file ncomms9299-s1.pdf]

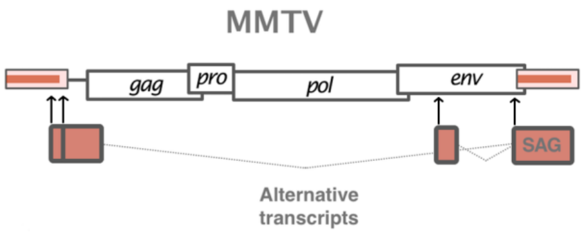

Supplementary Figure 1

Genome map of Mouse Mammary Tumour Virus (MMTV)

An approximately scaled diagram of the MMTV genome, representing the encoded genes as labelled boxes with LTRs at either end. The arrows indicate the approximate positions of alternative promoters, and alternative transcripts that also contain the *sag* gene in different conditions are represented below the genome map. The position of alternative transcripts were drawn according to[1].

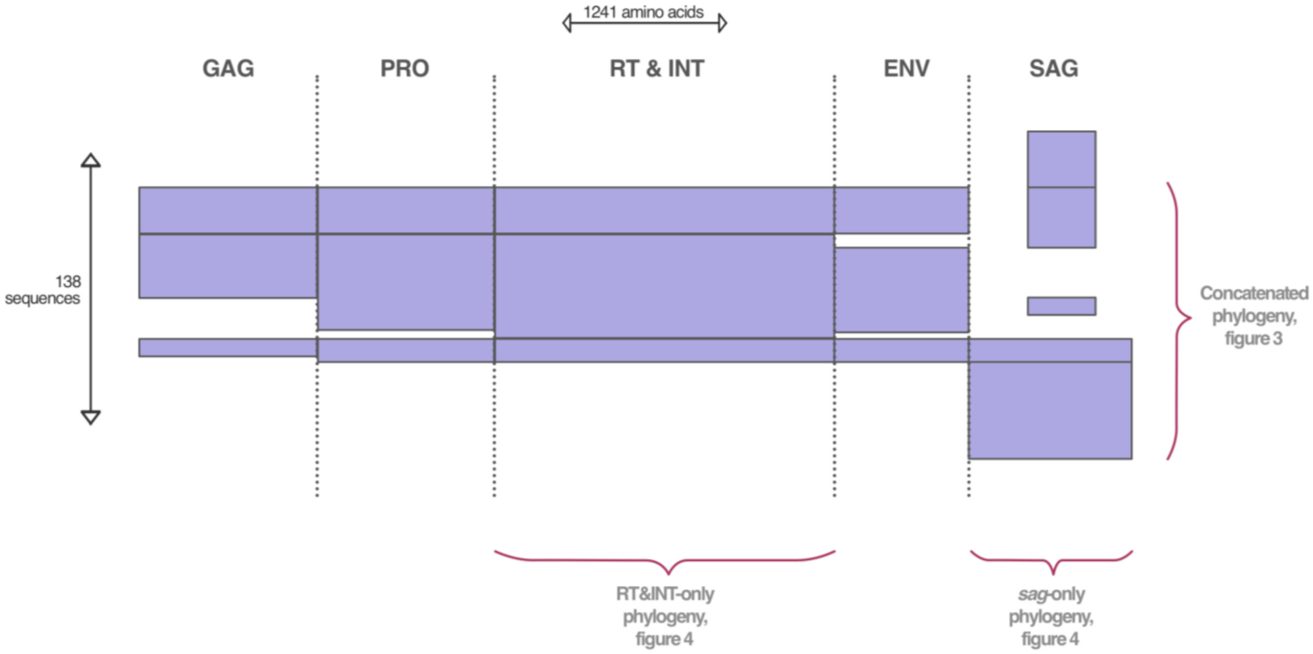

Supplementary Figure 2

Diagram of concatenated alignment of sequences used for phylogenetic analyses

The diagram depicts a schematic of the concatenated alignment created using mammalian ERVs and herpesviral *sag* sequences. Each of the three trees reported in the study were reconstructed using the subset of sequences indicated.

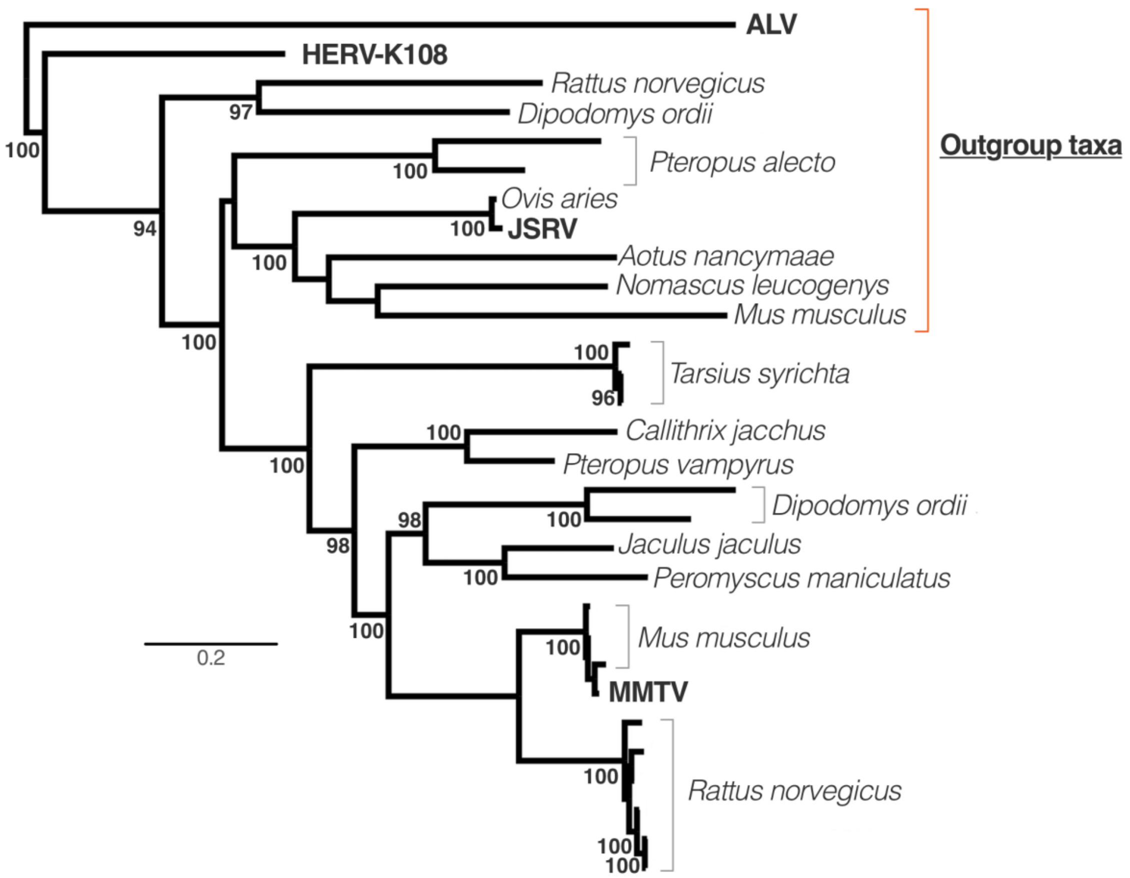

Supplementary Figure 3

Phylogenetic tree used to determine root of Figure 3 tree

This figure shows the phylogeny used to determine the placement of the root for the tree in Figure 3. It depicts a midpoint-rooted Bayesian tree of a polymerase alignment that included the outgroup sequence set with the pol-containing sequences in the concatenated tree. The numbers at each node represent posterior probability values (only those above 90% are shown). This tree shows that the in-group sequences are rooted at the branch leading to the *Tarsius syrichta* sequences. The scale bar indicated represents amino acid substitutions per site.

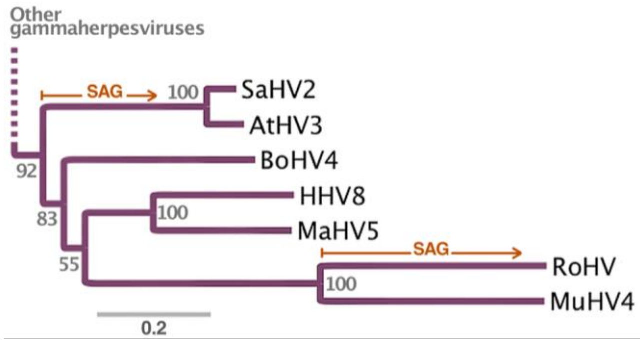

Supplementary Figure 4

Phylogenetic tree of rhadinoviruses using six conserved genes

The phylogeny shown is a subtree of a *gamaherpesvirinae* maximum likelihood phylogeny estimated using a concatenated alignment of 6 core genes (terminase, large tegument, uracil-DNA glycosylase, kinase, capsid protein and helicase). The scale bar indicated represents nucleotide substitutions per site. Values at each node represent bootstrap percentages as estimated by 1,000 non-parametric pseudoreplicates. Taxon abbreviation are RHVP: Rodent herpesvirus peru, MuHV4: Murid herpesvirus 4, AtHV3: Ateline herpesvirus 3, SaHV2: Saimirine herpesvirus 2, BoHV4: Bovine herpesvirus 4, HHV8: human herpesvirus 8, MaHV5: Macacine herpesvirus 5. The hypothesized model of *sag* acquisition is represented by a red arrow.

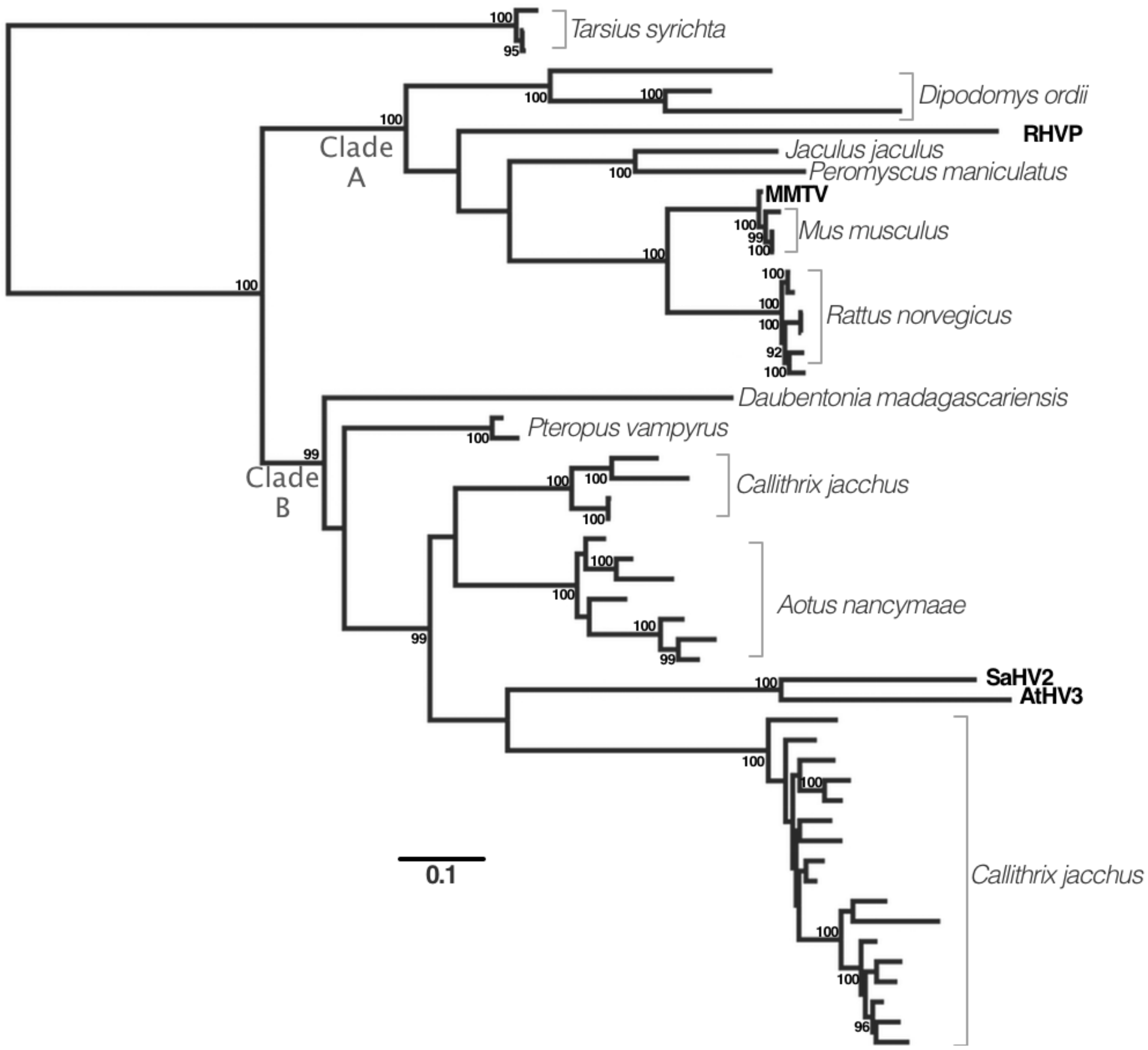

Supplementary Figure 5

Phylogenetic test of the monophyly of SaHV2 and AtHV3

A phylogeny of *gag, pro, pol, env* and *sag* genes with sites detected under positive selection removed from the alignment. This did not have any effect on the topology, although a slight change in branch length is observed as well as a slight increase of the posterior probability for some nodes (indicated at each node if above 90%). The scale bar indicated represents the number of amino acid substitutions per site.

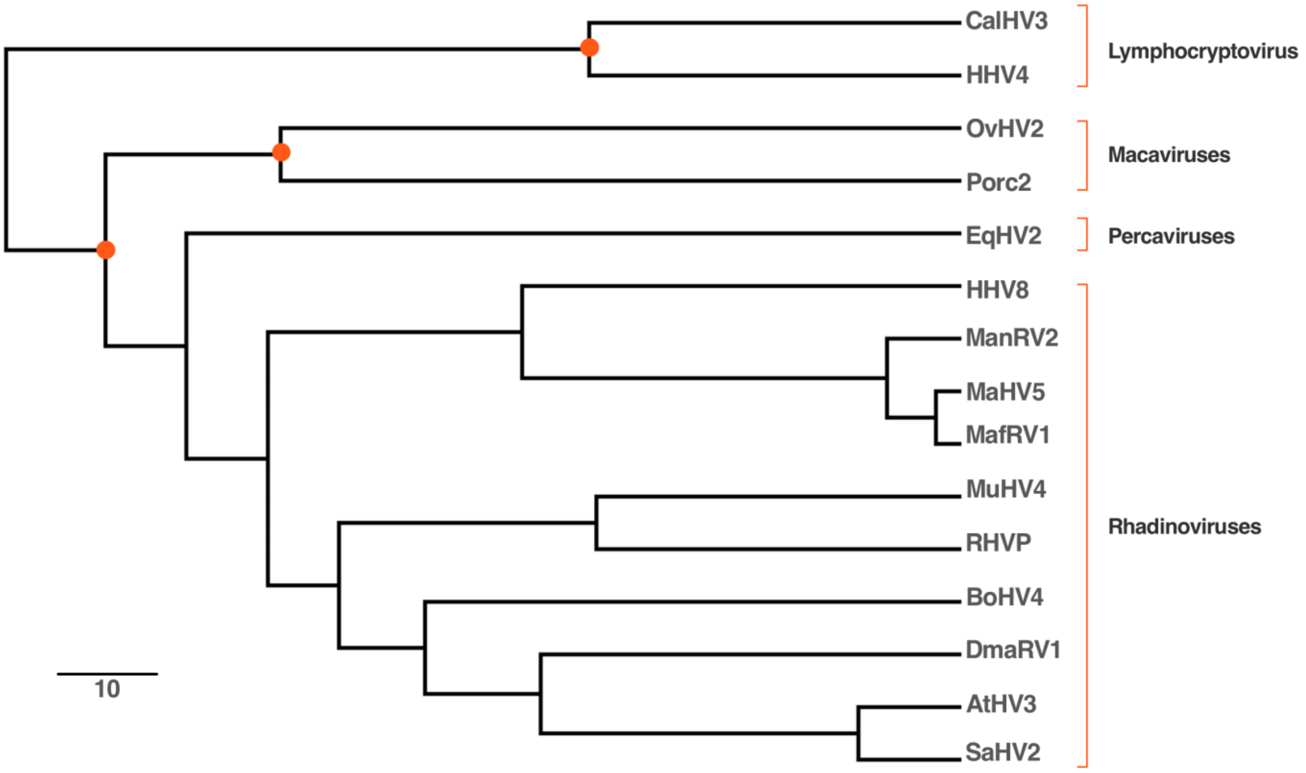

Supplementary Figure 6

Time-calibrated herpesvirus tree

The figure shown represents the time-calibrated phylogeny of herpesviruses estimated in BEAST, which was pruned down to the relevant tips for the tree in figure 5. The scale bar represents indicated is in million of years. Taxon abbreviation are RHVP: Rodent herpesvirus peru, MuHV4: Murid herpesvirus 4, AtHV3: Ateline herpesvirus 3, SaHV2: Saimirine herpesvirus 2, BoHV4: Bovine herpesvirus 4, HHV8: human herpesvirus 8, CalHV3: Callitrichine herpesvirus 3 , EqHV2: Equine herpesvirus 2, HHV4:Human herpesvirus 4, ManRV2: Macaca nemestrina rhadinovirus 2, MafRV1:Macaca fuscata rhadinovirus 1, OvHV2: Ovine herpesvirus 2, Porc2: Porcine lymphotropic herpesvirus 2, MaHV5: Macacine herpesvirus 5 , DmaRV1: Daubentonia madagascariensis radinovirus 1. (DmaRV1 sequences were identified in our previous publication[2]).

**Genus**	Aotus	Aotus	Aotus	Aotus	Aotus	Aotus	Aotus	Rhadinovirus	Rhadinovirus	Rhadinovirus	Alpharetrovirus	Class II ERV	Betarerovirus	Betarerovirus	Daubentonia	Callithrix	Callithrix	Callithrix	Pteropus
**Species**	nancymaae	nancymaae	nancymaae	nancymaae	nancymaae	nancymaae	nancymaae	saHV2	atHv3	roHV	ALV	HERV-K	JSRV	MMTV	madagascariensis	jacchus	jacchus	jacchus	vampyrus
**Accession**	JYKP01018661.1	JYKP01073282.1	JYKP01181974.1	JYKP01239066.1	JYKP01018659.1	JYKP01112511.1	JYKP01112426.1	NC_001350.1	NC_001987.1	NC_015049.1	NC_015116.1	NC_022518.1	NC_001494.1	NC_001503.1	AGTM012997089.1	ACFV01171011.1_2	ACFV01044201.1	ACFV01126740.1	ABRP01228684.1
gag											■	■	■	■					
pro											■	■	■	■					
pol											■	■	■	■					
env	■											■	■	■					
sag	■	■	■	■	■	■	■	■	■	■				■	■	■	■	■	■
**Genus**	Pteropus	Pteropus	Pteropus	Pteropus	Pteropus	Pteropus	Callithrix	Callithrix	Callithrix	Callithrix	Callithrix	Callithrix	Callithrix	Callithrix	Callithrix	Callithrix	Callithrix	Callithrix	Callithrix
**Species**	vampyrus	vampyrus	alecto	alecto	alecto	alecto	jacchus	jacchus	jacchus	jacchus	jacchus	jacchus	jacchus	jacchus	jacchus	jacchus	jacchus	jacchus	jacchus
**Accession**	ABRP01191652.1	ABRP01087430.1	ALWS01056471.1	ALWS01044416.1	ALWS01016527.1	ALWS01000833.1	ACFV01183383.1	ACFV01156521.1	ACFV01154330.1	ACFV01114849.1	ACFV01097500.1	ACFV01093596.1	ACFV01041432.1	ACFV01013848.1	ACFV01088484.1	ACFV01145903.1	ACFV01138800.1	ACFV01115463.1	ACFV01157657.1
gag																			
pro																			
pol																			
env																			
sag	■	■	■	■	■	■	■	■	■	■	■	■	■	■	■	■	■	■	■
**Genus**	Callithrix	Callithrix	Callithrix	Callithrix	Pteropus	Pteropus	Pteropus	Callithrix	erinaceus	erinaceus	erinaceus	Orycteropus	Orycteropus	erinaceus	erinaceus	erinaceus	erinaceus	erinaceus	erinaceus
**Species**	jacchus	jacchus	jacchus	jacchus	alecto	alecto	vampyrus	jacchus	europaeus	europaeus	europaeus	afer	afer	europaeus	europaeus	europaeus	europaeus	europaeus	europaeus
**Accession**	ACFV01083091.1	ACFV01070902.1	ACFV01067173.1	ACFV01173401.1	ALWS01040448.1	ALWS01070041.1	ABRP01272947.1	ACFV01171011.1	AMDU01213627.1	AMDU01124824.1	AMDU01056098.1	ALYB01353094.1	ALYB01313857.1	AMDU01014656.1	AANN01453706.1	AMDU01096953.1	AMDU01040360.1	AMDU01023674.1	AMDU01022785.1
gag							■	■											
pro							■	■											
pol							■	■											
env							■	■											
sag	■	■	■	■	■	■	■	■	■	■	■	■	■	■	■	■	■	■	■
**Genus**	erinaceus	erinaceus	erinaceus	Mus	Mus	Mus	Mus	Mus	Mus	Cricetulus	Cricetulus	Cricetulus	Cricetulus	Mus	Mus	Mus	Rattus	Rattus	Balaenoptera
**Species**	europaeus	europaeus	europaeus	musculus	musculus	musculus	musculus	musculus	musculus	griseus	griseus	griseus	griseus	musculus	musculus	musculus	norvegicus	norvegicus	acutorostrata
**Accession**	AMDU01015984.1	AANN01268976.1	AANN01232404.1	AHBB01142544.1	AEKQ02152907.1	AEKR01205738.1	CAAA01025638.1	AEKQ02014752.1	AEKQ02050583.1	AFTD01100812.1	AMDS01087150.1	AFTD01011754.1	AMDS01100063.1	AAHY01150974.1	AAHY01024305.1	CAAA01148393.1	AAHX01013382.1	AABR06028593.1	ATDI0100021
gag																			■
pro																			■
pol																			■
env																			■
sag	■	■	■	■	■	■	■	■	■	■	■	■	■	■	■	■	■	■	
**Genus**	Bos	Bos	Chrysochloris	Chrysochloris	Cricetulus	Dipodomys	Daubentonia	Elephantulus	Equus	Erinaceus	Erinaceus	Ictidomys	Leptonychotes	Lipotes	Lipotes	Loxodonta	Microtus	Mus	Mus
**Species**	indicus	taurus	asiatica	asiatica	griseus	ordii	madagascariensis	edwardii	caballus	europaeus	europaeus	tridecemlineatus	weddellii	vexillifer	vexillifer	africana	ochrogaster	musculus	musculus
**Accession**	AGFL01181997.1	AAFC03079735.1	AMDV01321751.1	AMDV01361218.1	APMK01250014.1	ABRO01007532.1	AGTM011639756.1	AMGZ01235975.1	AAWR02042168.1	AANN01090537.1	AMDU01141785.1	AAQQ0101014	APMU01073177.1	AUPI01040582.1	AUPI01145360.1	AAGU03002582.1	AHZW01004478.1	AAHY01071927.1	AEKQ02053596.1
gag	■	■	■	■	■	■	■	■	■	■	■	■	■	■	■	■	■	■	■
pro	■	■	■	■	■	■	■	■	■	■	■	■	■	■	■	■	■	■	■
pol	■	■	■	■	■	■	■	■	■	■	■	■	■	■	■	■	■	■	■
env	■	■	■	■	■	■	■	■	■	■	■	■	■	■	■	■	■		■
sag																		■	■
**Genus**	Mus	Mus	Mus	Mus	Peromyscus	Peromyscus	Myotis	Myotis	Ochotona	Odobenus	Orycteropus	Ovis	Peromyscus	Peromyscus	Procavia	Procavia	Rattus	Rattus	Rattus
**Species**	musculus	musculus	musculus	musculus	maniculatus	maniculatis	brandtii	lucifugus	princeps	rosmarus	afer	aries	maniculatus	maniculatus	capensis	capensis	norvegicus	norvegicus	norvegicus
**Accession**	AEKQ02185596.1	AEKQ02186966.1	CAAA01150610.1	CAAA01198845.1	AYHN01003103.1	EU204642.1	ANKR01221297.1	AAPE02008149.1	AAYZ01229114.1	ANOP01040725.1	ALYB01231012.1	AMGL01129945.1	AYHN01129821.1	AYHN01152242.1	ABRQ01011474.1	ABRQ01381071.1	AABR06024640.1	AABR06032972.1	AABR06085381.1
gag	■	■	■	■	■	■	■	■	■	■	■	■	■	■	■	■	■	■	■
pro	■	■	■	■	■	■	■	■	■	■	■	■	■	■	■	■	■	■	■
pol	■	■	■	■	■	■	■	■	■	■	■	■	■	■	■	■	■	■	■
env		■	■	■	■	■			■	■	■	■	■	■	■	■		■	■
sag				■	■												■	■	■
**Genus**	Rattus	Rattus	Rattus	Rattus	Sus	Tarsius	Tarsius	Tarsius	Tarsius	Tupaia	Tursiops	Aotus	Cricetulus	Dipodomys	Dipodomys	Dipodomys	Dipodomys	Echinops	Erinaceus
**Species**	norvegicus	norvegicus	norvegicus	norvegicus	scrofa	syrichta	syrichta	syrichta	syrichta	chinensis	truncatus	nancymaae	griseus	ordii	ordii	ordii	ordii	telfairi	europaeus
**Accession**	AABR06098027.1	AAHX01003470.1	AAHX01032432.1	AAHX01097670.1	AOCR01001337.1	ABRT010264555.1	ABRT010313192.1	ABRT02366711.1	ABRT02472330.1	ALAR01194664.1	ABRN01205561.1	AC201708	AMDS01143269.1	ABRO01000005.1	ABRO01000060.1	ABRO01005184.1	ABRO01009415.1	AAIY02121914.1	AMDU01169997.1
gag	■	■	■	■	■	■	■	■	■	■	■								
pro	■	■	■	■	■	■	■	■	■	■	■	■	■	■	■	■	■	■	■
pol	■	■	■	■	■	■	■	■	■	■	■	■	■	■	■	■	■	■	■
env	■		■	■		■	■	■	■	■	■	■	■		■	■	■	■	■
sag	■	■	■	■		■	■							■	■	■	■		
**Genus**	Jaculus	Mus	Peromyscus	Nomascus	Ochotona	Tarsius													
**Species**	jaculus	musculus	maniculatus	leucogenys	princeps	syrichta													
**Accession**	AKZC01311602.1	CAAA01158679.1	AYHN01193045.1	ADFV01049394.1	AAYZ01351999.1	ABRT010387126.1													
gag																			
pro	■	■			■	■													
pol	■	■		■	■	■													
env	■	■		■	■	■													
sag	■	■				■													

Supplementary Table 1

Details of sequences included in the concatenated alignment

For each sequence, the presence/absence of each gene in the alignment is denoted as a filled or blank cell, respectively. Species names and NCBI accession codes for the source contigs are indicated.

	Model	Free parameters	lnL	AIC
	One-ratio	1	-5884.97839	11771.95679
	Free-ratio	50	-5846.46793	11792.93586
Two-ratio	ωt,ωi	2	-5884.49031	11772.98062
	ωt,ωi=ω0	2	-5884.49031	11772.98062
	ωimh=ωth=ωt=ω0,ωia=ωim	2	-5884.49031	11772.98062
	ωimh=ωth=ωt=ω0,ωia=1,ωim	2	-5903.75545	11811.51089
	ωimh=ωth=ωt=ω0,ωia,ωib=1	2	-5910.25074	11824.50149
	ωth=ωt=ω0,ωia=ωib=1,ωimh	2	-5928.62362	11861.24724
	ωth=ωt=ω0,ωia=ωib,ωimh=1	2	-5885.83891	11775.67781
	ωt=ω0,ωia=ωib=ωimh,ωth=1	2	-5909.30869	11822.61738
	ω0,ωimh	2	-5884.97754	11773.95508
Three-ratio	ωimh=ωth=ωt=ω0,ωia,ωim	3	-5883.49986	11772.99972
	ωth=ωt=ω0,ωia,ωib,ωimh=1	3	-5884.92127	11775.84254
	ωth=ωt=ω0,ωia=ωib,ωimh	3	-5884.47495	11774.9499
	ωth=ωt=ω0,ωia=1,ωib,ωimh	3	-5900.66225	11807.32449
	ωth=ωt=ω0,ωia,ωib=1,ωimh	3	-5910.22637	11826.45273
	ωt=ω0,ωia=ωib,ωimh,ωth=1	3	-5909.30822	11824.61643
	ωt=ω0,ωia=ωib=ωimh,ωth	3	-5883.29022	11772.58043
	ωt=ω0,ωia=ωib,ωimh=ωth	3	-5883.28052	11772.56104
	ωt=ω0,ωia=ωib=ωimh=1,ωth	3	-5931.70421	11869.40842
	ωt=ω0,ωia,ωib=ωimh,ωth=1	3	-5907.98418	11821.96837
	ωt=ω0,ωia,ωib=ωimh=1,ωth	3	-5910.95674	11827.91348
	ωt=ω0,ωia=1,ωib=ωimh,ωth	3	-5900.45884	11806.91768
Multi-ratio	ωth=ωt=ω0,ωia,ωib,ωimh	4	-5883.48601	11774.97202
	ωt=ω0,ωia=ωib,ωimh,ωth	4	-5883.26153	11774.52306
	ωt=ω0,ωia,ωib=ωimh,ωth	4	-5882.59582	11773.19164
	ωt=ω0,ωia,ωib,ωimh,ωth	5	-5882.4447	11774.88939
	ωt=ω0,ωia,ωib,ωimh,ωtph	5	-5883.14786	11776.29572
	ωt=ω0,ωia,ωib,ωimh,ωtmh,ωtrhp	6	-5881.73242	11775.46484

Omega (ω) types

ω0	ω background
ωt	ω tips
ωia	ω internal clade A
ωib	ω internal clade B
ωimh	ω internal monkey herpesviruses
ωth	ω tips herpesviruses
ωtmh	ω tips monkey herpesviruses
ωtrhp	ω tip rodent herpesvirus peru

Supplementary Table 2

Maximum likelihood test of selection for main phylogeny in figure 3

Because there are a high number of ways that the 50 branches could be grouped, we only tested a series of biologically realistic hypotheses. The key labeled 'Omega (ω) types' indicates the symbols used to denote branch types. Although none of the models tested offered a significantly improved likelihood according to the likelihood ratio test, 16 of the hypotheses tested ranked higher than the free-ratio model according to the Akaike Information Criterion (AIC). The free-ratio model has a significantly better likelihood than the one-ratio, and underlined models are those that performed better than the free ratio test according to the AIC.

Pteropus alecto	*Pteropus vampyrus*	ω	estimated	fixed to one	P-value
ALWS01167045.1	ABRP01226597.1	1.2887	-1468.284955	-1468.362177	0.69432418
ALWS01038626.1	ABRP01001304.1	1.2344	-1705.567551	-1705.635601	0.712189342
ALWS01093920.1	ABRP01162448.1	1.0573	-1642.736517	-1642.739836	0.935065063
ALWS01169798.1	ABRP01020384.1	1.3079	-1805.989282	-1806.245837	0.473795651
ALWS01105028.1	ABRP01171138.1	1.1196	-1736.627038	-1736.654892	0.813412943
ALWS01104918.1	ABRP01114503.1	1.25	-1767.382375	-1767.515113	0.606382783
ALWS01103775.1	ABRP01158624.1	0.8619	-1734.607552	-1734.651535	0.766779068
ALWS01097461.1	ABRP01033558.1	1.1834	-1714.53515	-1714.610998	0.69691984
ALWS01078826.1	ABRP01079374.1	1.0683	-1795.940301	-1795.954459	0.866368052
ALWS01054040.1	ABRP01167243.1	0.7182	-1756.449987	-1756.721645	0.461061708
ALWS01009353.1	ABRP01087430.1	0.7691	-1690.028939	-1690.115408	0.677513806
ALWS01048320.1	ABRP01157105.1	1.0241	-1653.640581	-1653.641404	0.967637963
ALWS01055306.1	ABRP01174128.1	0.7097	-1588.763236	-1589.245527	0.32603514
ALWS01095756.1	ABRP01176227.1	2.8441	-1698.397185	-1699.612614	0.118967769
ALWS01010934.1	ABRP01014798.1	1.0723	-1667.086515	-1667.0953	0.894547793
ALWS01014665.1	ABRP01173071.1	0.7673	-1786.965452	-1787.173963	0.518426761
ALWS01020099.1	ABRP01184288.1	1.5341	-1665.587038	-1665.742479	0.577139459
ALWS01025422.1	ABRP01046600.1	1.2849	-1745.829143	-1745.974319	0.589995049
ALWS01032946.1	ABRP01053790.1	0.8868	-1666.080683	-1666.12071	0.777224245
ALWS01033198.1	ABRP01142907.1	1.1876	-1879.600579	-1879.726197	0.616206113
ALWS01037244.1	ABRP01034590.1	1.6693	-1716.619141	-1717.322582	0.235574689
ALWS01040448.1	ABRP01159955.1	0.4293	-1143.51778	-1144.625893	0.136566538
ALWS01040757.1	ABRP01170161.1	1.2433	-1698.251283	-1698.306593	0.739439376
ALWS01043129.1	ABRP01096911.1	1.0729	-1493.436316	-1493.458623	0.832715399
ALWS01046598.1	ABRP01032009.1	0.2822	-1662.960313	-1664.65104	0.065933865

Supplementary Table 3

Maximum likelihood test of selection for orthologous sequences in *Pteropus* species

The Table shows the accession numbers of the two species for each orthologous pair of LTRs and their respective Pairwise dN/dS (ω) values obtained through CODEML. The likelihood score for the estimated value was compared to the likelihood of fixing ω to one, and P-values obtained from a chi-squared test with one degree of freedom.

Supplementary References

1. Mustafa, F., Lozano, M. & Dudley, J. P. C3H mouse mammary tumor virus superantigen function requires a splice donor site in the envelope gene. *J. Virol.* **74,** 9431–40 (2000).
2. Aswad, A. & Katzourakis, A. The first endogenous herpesvirus, identified in the tarsier genome, and novel sequences from primate rhadinoviruses and lymphocryptoviruses. *PLoS Genet.* **10,** e1004332 (2014).
